# Non Hodgkin's lymphoma associated membranoproliferative glomerulonephritis: rare case of long term remission with chemotherapy: a case report

**DOI:** 10.4076/1757-1626-2-7201

**Published:** 2009-09-15

**Authors:** Hala Alshayeb, Barry M Wall

**Affiliations:** 1 University of Tennessee Health Science Center, Department of Internal Medicine, 956 Court Avenue, Room H314, Memphis, TN 38163, USA; 2 Veterans Affairs Medical Center and Department of Medicine, 1030 Jefferson Avenue, Suite G 410, Memphis, TN 38103, USA

## Abstract

**Introduction:**

Although membranoproliferative glomerulonephritis has been reported to occur in association with non-Hodgkin's lymphoma, information concerning the long term effects of treatment of non-Hodgkin's lymphoma on the associated membranoproliferative glomerulonephritis is limited.

**Case presentation:**

The current report describes a patient who presented with the abrupt onset of hypertension, mixed nephritic/nephrotic syndrome and acute renal failure. Kidney biopsy was consistent with membranoproliferative glomerulonephritis, type 1. Bone marrow biopsy performed in the evaluation of periaortic lymphadenopathy, hepatosplenomegaly, and thrombocytopenia confirmed the diagnosis of low grade B-cell non-Hodgkin's lymphoma. The patient's renal function improved and proteinuria resolved after initial treatment of non-Hodgkin's lymphoma with chemotherapy. During eleven years of follow up, membranoproliferative glomerulonephritis has remained in remission, as confirmed by repeatedly negative urinalyses, normal blood pressure and absence of clinical signs and symptoms suggestive of nephritic/nephrotic syndrome.

**Conclusion:**

Membranoproliferative glomerulonephritis has been known to be associated with both chronic lymphocytic leukemia and non-Hodgkin's lymphoma, particularly with B cell lymphocytic type non-Hodgkin's lymphoma. There is limited information available concerning the effects of treatment of non-Hodgkin's lymphoma on the progression of non-Hodgkin's lymphoma associated membranoproliferative glomerulonephritis. In the few reported cases we found, long term follow up after initial resolution of the membranoproliferative glomerulonephritis was lacking. This report presented a rare case of non-Hodgkin's lymphoma associated membranoproliferative glomerulonephritis, that continued to be in remission during eleven years of follow up after initial chemotherapy treatment of lymphoma.

## Introduction

Glomerulonephritis is a rare complication of hematological malignancies [1]-[3]. Chronic lymphocytic leukemia (CLL) and Non-Hodgkin's lymphoma (NHL) have been the most common hematologic malignancies associated with glomerular diseases [3]. Membranoproliferative glomerulonephritis (MPGN) has been the most commonly reported glomerular disease associated with CLL and has also been associated with Non-Hodgkin's lymphoma [1,3,4]. Although treatment of CLL with systemic chemotherapy has been previously associated with resolution of MPGN [2], information concerning the effects of treatment of NHL on the clinically associated MPGN, however, is limited and long term follow up is lacking in previously reported cases [3].

This report describes a patient who was diagnosed with MPGN and NHL simultaneously. MPGN and NHL resolved after treatment with chemotherapy. MPGN continued to be in remission during 11 years of follow up after treatment of NHL with chemotherapy.

## Case presentation

A 64 year old previously healthy white male construction worker presented with a four-week history of progressive shortness of breath, bilateral lower limb swelling, increased abdominal girth and a thirty pound weight gain. He was found to have new onset hypertension and an abnormal urinalysis.

On physical examination temperature was 98.6, pulse was 64 beat per minute and blood pressure was 208/104 mmHg. Fundoscopic exam was normal. Jugular veins were distended. Heart examination showed a displaced apical impulse. Breath sounds were decreased on the right side of the chest. The abdomen was remarkable for marked splenomegaly and shifting dullness. There was marked pretibial edema. No peripheral lymphadenopathy was detected.

Initial laboratory evaluation showed a white blood cell count of 7.2 ×10^9^/L, hematocrit 34%, and platelet count 99 × 10^9^/L. The serum creatinine was 2.9 mg/dl, and blood urea nitrogen was 37 mg/dl. Serum electrolytes were (in meq/l) sodium 143, potassium 4.3, chloride 112, and bicarbonate 22. Hepatic transaminases, alkaline phosphatase, and prothrombin time were normal. Total protein was 4.8 g/dl and albumin 2.6 g/dl. Urinalysis showed 3^+^ blood, 3^+^ protein with abundant granular casts and RBCS but no cellular casts. 24 hour urinary protein excretion was 28 g. Serum immunofixation electrophoresis, SPEP and UPEP were negative for monoclonal bands. Serum complement (C3) was 72.3 mg/dl (83-193) and C4 was 22.8 mg/dl (12-36). Rheumatoid factor was 299 IU (0-29). Tests for hepatitis B surface antigen, hepatitis C antibody, RPR, human immunodeficiency virus antibodies, streptozyme, and antinuclear antibodies were negative.

Chest radiographic study showed a right pleural effusion and a normal sized heart. Computerized tomography of the abdomen showed hepatosplenomegaly and periaortic lymphadenopathy. Doppler ultrasonography of the kidneys was normal.

### Pathologic findings

**Figure 1:**
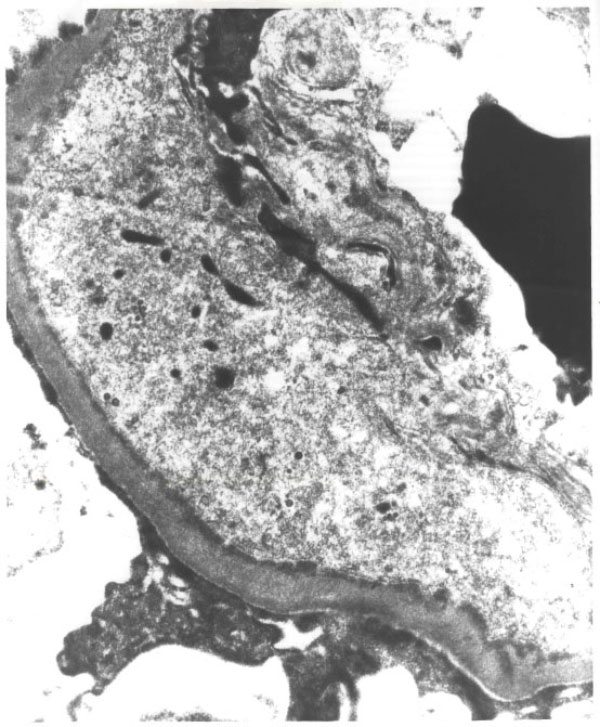
**Electron-microscopy: Subendothelial electron-dense deposits were seen along the basement membrane**. Mesangial electron-dense deposits were also detected. These histologic changes were consistent with MPGN, type 1.

A percutaneous renal biopsy was performed. Light microscopy showed increased cellularity in all glomeruli and accentuation of lobular architecture. Silver staining showed double contours within the capillary loops. Epithelial crescents were not present. Immunofluorescence studies showed granular staining in a lobular pattern for IgG, IgM, Kappa, and lambda. Electron microscopy showed subendothelial and mesangial fine electron dense deposits (Figure 1). These histologic changes were consistent with MPGN, type 1. There was no evidence of lymphomatous infiltration and there was no histological evidence of cyroglobulin deposition or amyloidosis.

Bone marrow aspiration and biopsy showed a hypercellular bone marrow (80%) with extensive infiltration of small B cell lymphoctyes. The B cells were positive for the B cell marker CD20 and negative for T cell markers CD5 and CD3. The final diagnosis was low grade B cell NHL.

### Long-term follow up

After initial treatment with six cycles of cyclophosphamide, vincristine, and prednisone. The patient improved symptomatically and his blood pressure normalized. Serum creatinine, which initially peaked at 7.1 mg/dl, decreased dramatically to a baseline level of 1.4 - 1.7 mg/dl. Urine protein excretion decreased to <300 mg/24hr. Microscopic hematuria resolved. Serum albumin increased to 3.5-4.0 g/dl with resolution of the proteinuria. The anemia and thrombocytopenia resolved. Repeated computed tomography of the abdomen at intervals of six and 12 months showed improvement in splenomegaly and periaortic lymphadenopathy. A repeat bone marrow biopsy showed no evidence for NHL. During long term follow up, the patient has had two relapses of NHL confirmed by bone marrow biopsies. The first relapse was treated with six cycles of fludarabine and mitoxantrone and the second with rituximab. Subsequent bone marrow biopsy showed no evidence for lymphoma, and computed tomography of the abdomen and pelvis showed resolution of lymphadenopathy with interval improvement in splenomegaly. In each relapse there was a transient increase in the serum creatinine concentration, up to 2 mg/dl, that was attributed to factors other than MPGN. Urinalysis remained normal. The patient continues to be in complete remission with respect to his MPGN and NHL, approximately 11 years following the initial diagnosis.

## Discussion

The current report describes a patient with previously normal kidney function who developed rapidly progressive membranoproliferative glomerulonephritis (MPGN, type I) in association with low grade non-Hodgkin's lymphoma. None of the more commonly associated clinical entities with MPGN type I were identified. Subsequent chemotherapy led to remission of NHL and to complete and persistent resolution of glomerulonephritis during eleven years of follow up.

Glomerular diseases have been known to be associated with both chronic lymphocytic leukemia and non-Hodgkin's lymphoma, particularly with B cell lymphocytic type NHL [1,2,4,5]. Membranoproliferative glomerulonephritis has been the most commonly reported histological diagnosis. Minimal change disease, focal segmental glomerulosclerosis, membranous glomerulopathy, mesangial proliferative glomerulonephritis, IgA nephropathy, and monoclonal immunoglobulin deposit diseases have also been reported. Glomerular involvement has preceded, coexisted with or even followed the diagnosis of lymphoma by several years.

The pathogenesis of non-Hodgkin's lymphoma-associated glomerulonephritis is poorly understood [5]-[7]. A current concept of the pathogenesis of paraneoplastic glomerulonephritis focuses on immune complexes containing tumor antigens, which are deposited in the glomeruli [6,7]. Autoimmune mechanisms and T- lymphocyte dysfunction have also been postulated to play a role in pathogenesis [5]-[7]. Glomerulonephritis may also be related to the production of cryoglobulins and/or immunoglobulin synthesis (paraprotein production) by the secretory B cell clone [8]. Chronic hepatitis C virus infection is currently the most common cause of secondary MPGN and is often associated with cryoglobulinemia. Although HCV serology was negative in this patient, any condition associated with persistent immunological stimulation can result in secondary cryoglobulinemia. Cryoglobulins were not directly measured in our patient; rheumatoid factor activity, however, was detected at the time of the diagnosis of MPGN and was undetectable after the resolution of glomerulonephritis such that cryoglobulinemia cannot be excluded as a pathogenic mechanism in this patient. Para proteins were not detected in our patient, and there was no electron microscopic evidence of fibrillary glomerulonephritis, immunotactoid glomerulopathy, or lymphomatous infiltration of the kidney [9,10].

There is limited information available concerning the effects of treatment of NHL on the progression of NHL associated MPGN. Moulin, *et al.* reported one patient with NHL who developed complete remission of MPGN after treatment with chlorambucil and a second patient who showed significantly improved renal function after treatment with MOPP (nitrogen mustard, vincristine, procarbazine, prednisone). Stokes, *et al.* described a patient with low-grade B-cell lymphoma and MPGN, who responded by improvement in renal function and diminution of proteinuria after treatment with oral prednisone.

MPGN in our patient continues to be in clinical remission with normal kidney function and normal urinalysis 11 years after the initial treatment of NHL despite two relapses of NHL that were effectively treated with additional chemotherapy. In each relapse of NHL there was a transient increase in serum creatinine concentration that was attributed to factors other than MPGN, as suggested by repeatedly normal urinalyses, normal blood pressure and the absence of clinical signs and symptoms associated with nephritic/nephrotic syndrome.

## Conclusion

The findings of normal renal function prior to the development of NHL in our patient, the development of MPGN temporally associated with the onset of NHL, the absence of other known causes of secondary MPGN, and remission of MPGN following NHL treatment strongly support a pathogenetic mechanism involving cryoglobulins or tumor antigens in the development of MPGN, type I. Early treatment of NHL with chemotherapy can be associated with long term remission of the associated MPGN as described in this report which was rarely reported in literature. This case underlines the importance of aggressive treatment of malignancy in patients with glomerulonephritis and of glomerulonephritis in patients with malignancy.

## Abbreviations

HCV: hepatitis C virus; MPGN: membranoproliferative glomerulonephritis; NHL: non-Hodgkin's lymphoma; RBCS: red blood cells; RPR: reactive plasma reagent; SPEP: serum protein electrophoresis; UPEP: urine protein electrophoresis.

## Consent

Written informed consent was obtained from the next-of-kin of the patient for publication of this case report and any accompanying images. A copy of the written consent is available for review by the Editor-in-Chief of this journal.

## Competing interests

The authors declare that they have no competing interests.

## Authors' contributions

HA and BMW secured the case, conducted the literature review, and participated in the preparation of the manuscript. Both authors read and approved the final manuscript.
